# Mutations in *PNKP* Cause Recessive Ataxia with Oculomotor Apraxia Type 4

**DOI:** 10.1016/j.ajhg.2015.01.005

**Published:** 2015-03-05

**Authors:** Jose Bras, Isabel Alonso, Clara Barbot, Maria Manuela Costa, Lee Darwent, Tatiana Orme, Jorge Sequeiros, John Hardy, Paula Coutinho, Rita Guerreiro

**Affiliations:** 1 Department of Molecular Neuroscience, Institute of Neurology, University College London, Queen Square, London WC1N 3BG, UK; 2 Center for Predictive and Preventive Genetics, Institute for Molecular and Cell Biology, University of Porto, Porto 4150-180, Portugal; 3 Unidade de Investigação Genética e Epidemiológica em Doenças Neurológicas Research Group, Institute for Molecular and Cell Biology, University of Porto, Porto 4150-180, Portugal; 4 Instituto de Investigação e Inovação em Saúde, University of Porto, Porto 4150-180, Portugal; 5 Hospital Pedro Hispano, Unidade Local de Saúde de Matosinhos, Matosinhos 4454-509, Portugal

## Abstract

Hereditary autosomal-recessive cerebellar ataxias are a genetically and clinically heterogeneous group of disorders. We used homozygosity mapping and exome sequencing to study a cohort of nine Portuguese families who were identified during a nationwide, population-based, systematic survey as displaying a consistent phenotype of recessive ataxia with oculomotor apraxia (AOA). The integration of data from these analyses led to the identification of the same homozygous *PNKP* (polynucleotide kinase 3′-phosphatase) mutation, c.1123G>T (p.Gly375Trp), in three of the studied families. When analyzing this particular gene in the exome sequencing data from the remaining cohort, we identified homozygous or compound-heterozygous mutations in five other families. PNKP is a dual-function enzyme with a key role in different pathways of DNA-damage repair. Mutations in this gene have previously been associated with an autosomal-recessive syndrome characterized by microcephaly; early-onset, intractable seizures; and developmental delay (MCSZ). The finding of *PNKP* mutations associated with recessive AOA extends the phenotype associated with this gene and identifies a fourth locus that causes AOA. These data confirm that MCSZ and some forms of ataxia share etiological features, most likely reflecting the role of PNKP in DNA-repair mechanisms.

## Main Text

Hereditary autosomal-recessive cerebellar ataxias (ARCAs) are rare neurodegenerative disorders that are clinically and genetically very heterogeneous and are characterized by cerebellar ataxia that is frequently associated with peripheral sensorimotor neuropathy. Ataxia with oculomotor apraxia (AOA) is a subgroup involving cerebellar ataxia, sensorimotor axonal neuropathy, oculomotor apraxia, and extrapyramidal features. Chromosomal instability, immunodeficiency, and sensitivity to ionizing radiations, all usually observed in persons with ataxia telangiectasia (AT) and AT-like disorders, are absent in individuals with AOA.^1^ Early-onset recessive ataxia (AOA1 or EAOH [MIM 208920]) is a progressive syndrome associated with hypoalbuminemia and elevated levels of cholesterol and is caused by mutations in *APTX* (aprataxin).^1–3^ Autosomal-recessive spinocerebellar ataxia 1 (AOA2 or SCAR1 [MIM 606002]), a progressive ataxia,^4^ occurring later than AOA1, is characterized by increased alpha-fetoprotein levels and is caused by mutations in *SETX* (senataxin).^5^ A third gene, *PIK3R5* (phosphoinositide-3-kinase, regulatory subunit 5; AOA3 [MIM 615217]), has been identified in a consanguineous Saudi Arabian family whose affected members have clinical features similar to those of individuals with AOA2.^6^

Next-generation sequencing technologies have been remarkably useful for the identification of novel genes causing Mendelian diseases. At the same time, these recently developed technologies have allowed the expansion of phenotypes, particularly in various neurological diseases. The association of the same molecular event with a large-spectrum phenotype or very different phenotypes can be explained by several factors, one being the pleiotropism associated with many genes.^7^

We used exome sequencing and homozygosity mapping to study a series of nine Portuguese families affected by early-onset recessive AOA. These families were identified during a nationwide, population-based, systematic survey of hereditary ataxias and spastic paraplegias. Performed in Portugal from 1994 to 2004, this survey used multiple sources of information to identify affected persons in the community and health-care settings. Detailed methods of the survey are described elsewhere.^8^ All individuals studied here presented established clinical criteria for AOA,^1^ and samples were collected after receipt of written informed consent from participants. This study used only de-identified, previously collected DNA samples that were stored at the authorized Center for Predictive and Preventive Genetics, Institute for Molecular and Cell Biology biobank and database.

After previous exclusion of mutations in the genes known to be directly associated with this phenotype (*APTX*, *SETX*, and *PIK3R5*), we performed whole-genome genotyping in ten individuals from seven families (nine affected and one unaffected). We used Illumina OmniExpress Beadchips, which assay over 700,000 markers, as per the manufacturer’s instructions. We used Illumina GenomeStudio for initial data analysis and quality control, which led to the exclusion of three samples (two from family 2 and one from family 6) from further analysis involving whole-genome genotyping data. Runs of homozygosity (ROH) were identified with PLINK v.1.07^9^ on the basis of having a size greater than 1.5 Mb and a minimum of 50 SNPs per region. We scanned the genome for regions of homozygosity by using a sliding window of 50 SNPs and allowing at most two missing genotypes and one heterozygote call per ROH. We focused the initial homozygosity mapping on pedigrees with reported consanguinity (families 5 and 7). The only homozygous region, a region >1.5 Mb and shared by all affected individuals, was on chromosome 19 (49,506,390–51,400,356) (Figure S1). Upon analysis of the other families, we found that the proband of family 8 also shared a large homozygous tract overlapping this region (chr19: 49,425,838–53,225,722).

We performed exome sequencing in 16 individuals from eight families (12 affected and four unaffected). For this analysis, we prepared genomic DNA according to Illumina’s TruSeq Sample Preparation v.3 and performed the exome capture with Illumina’s TruSeq Exome Enrichment according to the manufacturer’s instructions. Sequencing was performed on an Illumina HiSeq2500 with 100-bp paired-end reads. Following quality control procedures, samples yielded between 10.1 and 12.7 Gb of high-quality, aligned data. This amount of data was the result of mean target coverage between 66.39× and 88.5×, 93.5%–94.6% of targets’ being covered at greater than or equal to 10×, and less than 0.2% of targets’ not being covered even once. We performed sequence alignment and variant calling against the reference human genome (UCSC Human Genome Browser hg19) by using the Burrows-Wheeler Aligner^10^ and the Genome Analysis Toolkit.^11,12^ Prior to variant calling, PCR duplicates were removed with the Picard software. On the basis of the hypothesis that the mutation was rare, we excluded all common SNPs (MAF > 5%) identified in dbSNP v.137 and in our in-house database of sequencing data for other diseases (n > 2,000). Given the apparent autosomal-recessive mode of inheritance in the three families (families 5, 7, and 8) and the fact that two parental pairs were consanguineous, we focused on homozygous variants located in the shared ROH. One *PNKP* mutation, c.1123G>T (p.Gly375Trp; RefSeq accession number NM_007254.3), was present in homozygosis in all affected individuals and absent in all unaffected ones. Expanding this analysis to the other studied families, we found the same mutation in homozygosis in family 2; probands in families 1, 3, 4, and 6 were compound heterozygotes for different mutations in the same gene. The proband in family 6 was tested by Sanger sequencing, and no causative mutations were found in this gene in family 9. We also used Sanger sequencing to confirm all mutations identified by exome sequencing, establish compound heterozygosity, and verify intrafamilial segregation. We performed PCR amplifications and purified the resulting products with ExoSAP-IT (USB), then performed direct Sanger sequencing of both strands with BigDye Terminator v.3.1 chemistry v.3.1 (Applied Biosystems) and an ABI 3730XL Genetic Analyzer (Applied Biosystems). Sequencing traces were analyzed with Sequencher software v.4.2 (Gene Codes).

**Figure 1:**
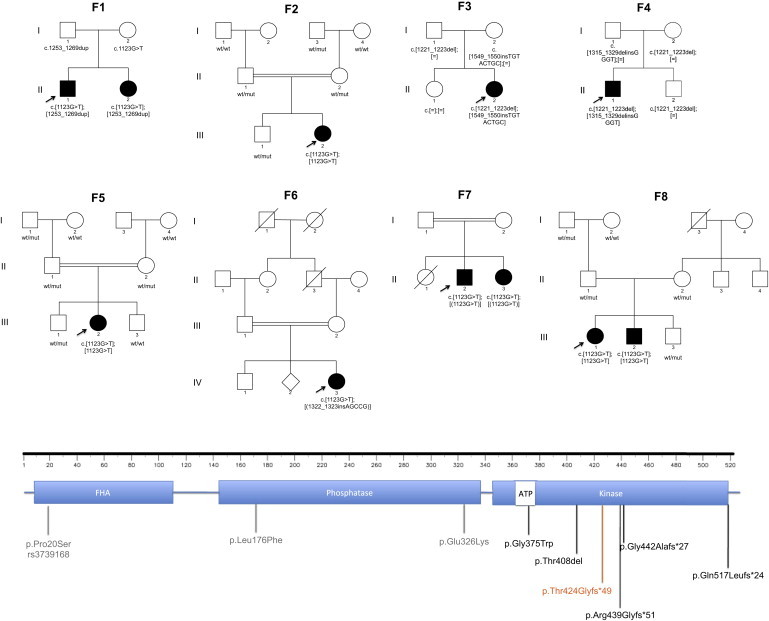
Pedigrees of the Portuguese Families In which AOA-Affected Members Had *PNKP* Mutations and Protein Representation with the Location of the Variants Found to Be Associated with Different Phenotypes Black symbols represent individuals affected by AOA. An arrow indicates the index individual in each family. Mutation segregation with disease is indicated underneath each symbol; all unaffected family members carry either a single variant (wt/mut) or no variants (wt/wt), and all affected individuals harbor mutations in homozygosity or compound heterozygosity. In the lower panel, PNKP (RefSeq NP_009185.2) is represented with the depicted regions as predicted by UniProtKB (Q96T60). Variants found in this study are represented in black and are all located in or adjacent to the kinase region of the protein. Variants previously identified in individuals with MCSZ are represented in gray. An additional 17-bp intron 15 deletion was reported in one family, which is not represented here.^13^ The variant p.Thr424Glyfs^∗^49 (in orange) has been previously associated with MSCZ^13^ and with progressive cerebellar atrophy and polyneuropathy.^14^ The same change was identified in compound heterozygosity with p.Gly375Trp in family 1. Targeted resequencing has also identified the p.Pro20Ser (c.58G>A) variant in an individual with epileptic encephalopathy;^15^ this variant was considered to be pathogenic in the homozygous state but had previously been identified in the Exome Variant Server (overall MAF of 0.9%) and dbSNP databases and classified as benign in ClinVar.

The main clinical features of the 11 AOA-affected persons (four males and seven females) who were found to have *PNKP* mutations are presented in Table 1. All of them were either homozygotes or compound heterozygotes for *PNKP* mutations (Table 2 and Figure 1). Age at onset ranged from 1 to 9 years; the mean was 4.3 ± 2.3 years. Most individuals exhibited dystonia as their first symptom; this was so prominent that many of these individuals underwent diagnostic procedures for extrapyramidal disorders. In all cases, dystonia spontaneously attenuated during the course of the disease. The second most common symptom was ataxia, followed by oculomotor apraxia. All individuals had signs of polyneuropathy with early, generalized areflexia. Distal-muscle wasting and weakness led to tetraplegia and short atrophic hands and feet. Loss of the ability to walk occurred 7–21 years after disease onset, and most individuals were wheelchair bound by adolescence. Cognitive impairment was present in seven persons, two of whom were severely demented. Alpha-fetoprotein, albumin, and cholesterol levels were highly variable in this cohort; alpha-fetoprotein levels were increased in five individuals, albumin was decreased in six, and cholesterol was elevated in five cases, all of which was determined at later stages of disease progression. In all 11 individuals, brain MRIs revealed cerebellar atrophy.

Mutations in *PNKP* were originally found to cause early infantile epileptic encephalopathy-10 (EIEE10 [MIM 613402]), characterized by microcephaly, seizures, and developmental delay.^13^ More recently, this gene was associated with cerebellar atrophy in two Dutch siblings who had progressive debilitating polyneuropathy, microcephaly, severe intellectual disability, and mild epilepsy.^14^ No ocular signs were described in those Dutch cases. None of the individuals we studied had microcephaly or epilepsy; cognitive impairment was observed in most but not all individuals (although it progressed to severe dementia in two cases).

These families were identified through a population-based nationwide survey for hereditary ataxias, to the best of our knowledge the largest ever performed. We can thus conclude that AOA4 is the most frequent form of AOA in the Portuguese population (it affects nearly 40% of the families identified) and the most frequent recessive ataxia in this population, after Freidreich ataxia.^8^ Mean age at onset (4.3 years) for individuals with AOA4 is closer to that of individuals with AOA1 (6.9 ± 4.7 years) than to that of those with AOA2 (14.6 ± 3.4), although it is lower than both.^16,17^ Clinical presentation, with marked extrapyramidal manifestations and its rapid progression, also more closely resembles that of AOA1 than that of AOA2. Additionally, in individuals with AOA4, albumin may be low or normal and cholesterol levels are normal or high, a similar situation to what is seen in individuals with AOA1 after some years of disorder progression. On the other hand, **α**-fetoprotein was elevated in some individuals with AOA4, which seems to always be the case in those with AOA2.

The different phenotypes so far associated with mutations in *PNKP* do not seem to relate to either the type or the location of the mutation (Figure 1). PNKP is a modular protein with three domains: an amino-terminal fork-head-associated (FHA) domain, which mediates interactions with the scaffold proteins XRCC1 and XRCC4; a DNA phosphatase domain; and a DNA kinase domain.^18^ The same homozygous variant, p.Thr424Glyfs^∗^49 (c.1253_1269dup), located in the kinase domain of the protein, has been found in individuals with progressive cerebellar atrophy and in individuals with MCSZ (an autosomal-recessive syndrome characterized by microcephaly, early onset, intractable seizures, and developmental delay).^13,14^ We have now identified this same variant in compound heterozygosity with p.Gly375Trp in family 1. Missense substitutions in the FHA and phosphatase domains of the protein have only been associated with developmental syndromes. In most affected individuals, MCSZ was found to be caused by the p.Glu326Lys (c.1123G>T) variant in homozygosity.^13^ In the present study, variants found to be associated with AOA were all located in, or adjacent to, the kinase region of the protein. The position of the most frequent variant, p.Gly375Trp, in this cohort is highly conserved across species (Figure S2) and is located in a potential ATP nucleotide-binding domain (amino acids 372–379) within the kinase region. Additionally, several in-silico-prediction software programs rate this variant as deleterious (SIFT score = 0; PolyPhen score = 0.999; CADD_phred = 18.51).^19^

A functional assessment of the impact of MCSZ-associated *PNKP* mutations showed that the studied variants reduced the cellular stability and the level of PNKP; some ablated cellular DNA 5′ kinase activity, and all studied variants reduced cellular 3′ phosphatase activity.^20^ The structural unit of the wild-type protein is stabilized by the anchoring of the kinase domain to the phosphatase domain via interactions between the five C-terminal residues, Q517–G521, and the interdomain linker and phosphatase domain.^18,20^ The deletion found in family 3, although adjacent to the kinase region of the protein, probably affects these interactions and thereby has consequences for protein conformation and function. It is possible that the different phenotypes associated with the different mutations result from defects in the phosphatase or kinase activities of the protein or from conformational protein alterations that lead to deficient protein-protein interactions or substract recognition. It is also plausible that the different phenotypes are the result of an additional genetic-variation load.^21^ PNKP has important roles in multiple pathways involved in DNA-damage repair, including single-strand breaks (SSBs) and double-strand breaks (DSB).^14,21,22^ The cellular response to these breaks involves a complex signaling network and the key activator of this network is the serine/threonine protein kinase ATM (AT mutated), which has been shown to target PNKP.^23,24^ Mutations in *ATM* are known to cause AT, another autosomal-recessive ataxia. Mutations in other ataxia-related genes, such as *TDP1* (tyrosyl-DNA phosphodiesterase-1, associated with spinocerebellar ataxia with axonal neuropathy-1 (SCAN1) [MIM 607250]) and *APTX*, also result in defects in SSB repair. In this way, the molecular involvement of PNKP in these different pathways, and the possible compensation by related proteins, might also contribute to the phenotypic differences associated with mutations in this gene.^22^ A STRING analysis supported this possibility, showing that PNKP interacts with proteins involved in neurodegenerative diseases and microcephaly syndromes.^14^ The phenotypes associated with PNKP mutations could also be influenced by gene-environment interactions. It has been recently demonstrated that physiologically and environmentally relevant doses of cadmium and copper can inhibit both the phosphatase and kinase activities of PNKP.^25^

In this study, we identified homozygous or compound-heterozygous *PNKP* mutations in eight of the nine Portuguese families we studied (with a total of 11 affected individuals), suggesting that, in Portugal, mutations in *PNKP* are the most frequent cause of AOA. However, additional studies are needed to determine whether this is also the case in other populations. Our results also extend the phenotype associated with mutations in this gene. Future studies clarifying PNKP regulation and interaction with other repair enzymes might help explain this phenotypic variability.

## Figures and Tables

**Table 1:** Clinical, Biochemical, and Imaging Features of the Individuals Carrying *PNKP* Mutations

	**Family 1**		**Family 2**	**Family 3**	**Family 4**	**Family 5**	**Family 6**	**Family 7**		**Family 8**	
	**P1**	**P2**	**P3**	**P4**	**P5**	**P6**	**P7**	**P8**	**P9**	**P10**	**P11**
Consanguinity	−		yes	−	−	yes	yes	yes		−	
Gender	male	female	female	female	male	female	female	female	male	female	male
Age at onset (years)	5	9	6	3	1	3	4	4	7	2	3
First sign	dystonia	dystonia	dystonia	ataxia	dystonia	ataxia	ataxia	OMA	OMA	dystonia	dystonia
More prominent sign	neurop.	neurop.	neurop.	neurop.	neurop.	neurop.	neurop.	neurop.	neurop.	neurop.	neurop.
OMA	+++	+++	+	++	+	+	++	+++	+++	+++	+++
Age in wheelchair (years)	18	22	18	20	NA	15	25	15	14	20	15
Dystonia	+	+	+	+	++	+	−	−	−	++	++
Cognitive impairment	+	+	++	+	NA	−	−	−	+	++	+
Motor deficit	+++	++	+++	+++	+++	+++	+++	+++	+++	+++	+++
Decreased vibration sense	++	+	NA	++	NA	++	+++	++	++	NA	++
Pyramidal signs	−	−	+	−	−	−	−	−	−	+	−
Obesity	−	−	+	+	−	−	+	−	−	++	−
MRI findings	CA	CA	CA	CA	CA	CA	CA	CA	CA	CA	CA
Other features	BSA	BSA	dem.	NA	NA	NA	NA	NA	NA	dem.	BSA
α-fetoprotein levels	1.5 N	1.5 N	1.5 N	N	N	1.5 N	4 N	N	N	N	N
Albumin levels	N	N	↓	↓	↓	↓	N	NA	NA	↓	↓
Cholesterol levels	N	N	N	↑	↑	N	↑	NA	NA	↑	↑

Abbreviations and symbols are as follows: P1–P11, individuals 1–11, respectively; +, present; ++, moderate; +++, severe; −, absent; ↑, increased level; ↓, decreased level; N, normal level; NA, not available; CA, cerebellar atrophy; BSA, brainstem atrophy; OMA, oculomotor apraxia; neurop., neuropathy; dem., dementia.

**Table 2:** *PNKP* Mutations that Caused AOA in Affected Members of the Eight Portuguese Families in Whom Mutations Were Found

**Family**	**cDNA (RefSeq NM_007254.3)**	**Protein (RefSeq NP_009185.2)**
1	c.[1123G>T];[1253_1269dupGGGTCGCCATCGACAAC]	p.[(Gly375Trp)];[(Thr424Glyfs^∗^49)]
2	c.[1123G>T];[1123G>T]	p.[(Gly375Trp)];[(Gly375Trp)]
3	c.[1221_1223del];[1549_1550insTGTACTGC]	p.[(Thr408del)];[(Gln517Leufs^∗^24)]
4	c.[1221_1223del];[1315_1329delinsGGGT]	p.[(Thr408del)];[(Arg439Glyfs^∗^51)]^a^
5	c.[1123G>T];[1123G>T]	p.[(Gly375Trp)];[(Gly375Trp)]
6	c.[1123G>T(;)1322_1323insAGCCG]	p.[(Gly375Trp(;)Gly442Alafs^∗^27)]
7	c.[1123G>T];[(1123G>T)]	p.[(Gly375Trp)];[(Gly375Trp)]
8	c.[1123G>T];[1123G>T]	p.[(Gly375Trp)];[(Gly375Trp)]

a One of the allelic mutations in F4 is formed by a complex allele that was initially not properly identified. Figure S3 shows the detailed view of the alignment at that position, clearly displaying the complex allele.

## Supplemental Data

Document S1. Figures S1–S3

Document S2. Article plus Supplemental Data

## Web Resources

The URLs for data presented herein are as follows:

dbSNP, http://www.ncbi.nlm.nih.gov/projects/SNP/
OMIM, http://www.omim.org/
Picard, http://picard.sourceforge.net/
UCSC Human Genome Browser, http://genome.ucsc.edu/cgi-bin/hgGateway
