# Supplementary material for: Mutations in PNKP Cause Recessive Ataxia with Oculomotor Apraxia Type 4
Source: Am J Hum Genet. 2015 Mar 5;96(3):474–9. doi: 10.1016/j.ajhg.2015.01.005 (PMC4375449; doi:10.1016/j.ajhg.2015.01.005)
Supplement: Document S1. Figures S1–S3 [file mmc1.pdf]

The American Journal of Human Genetics

Supplemental Data

Mutations in *PNKP* Cause Recessive Ataxia with Oculomotor Apraxia Type 4

Jose Bras, Isabel Alonso, Clara Barbot, Maria Manuela Costa, Lee Darwent, Tatiana Orme, Jorge Sequeiros, John Hardy, Paula Coutinho, Rita Guerreiro

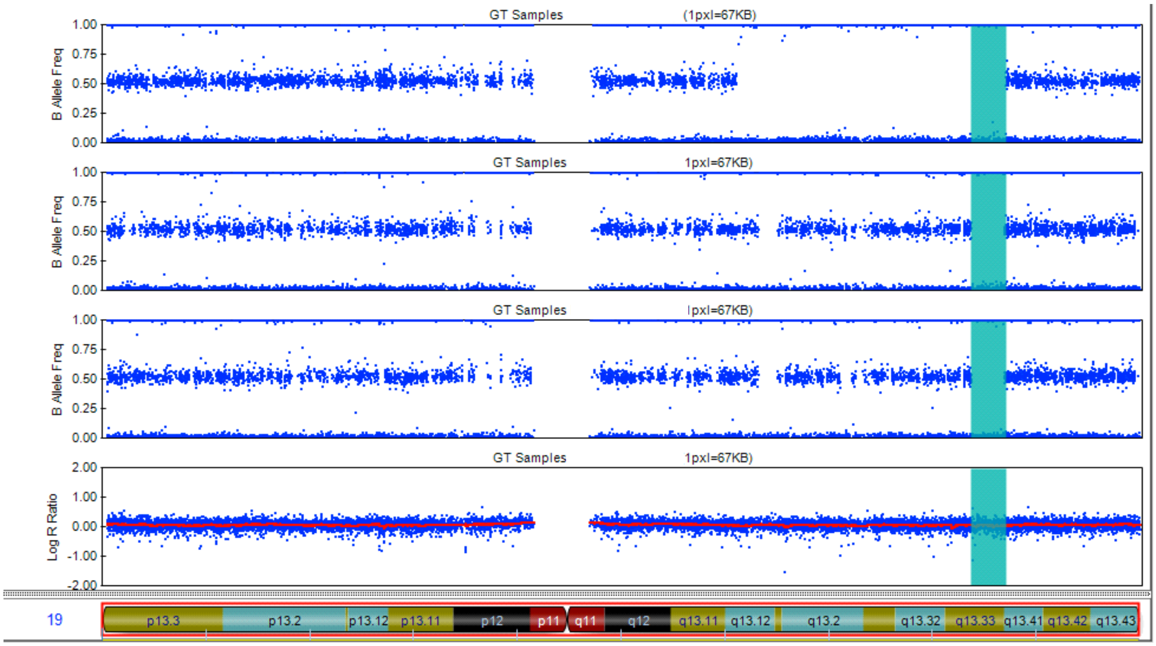

Figure S1. Representation of the homozygosity mapping results for the families with reported consanguinity.

The results from the Illumina Infinium whole-genome genotyping assay, as visualized in GenomeStudio, are shown for two families with reported consanguinity. The figure shows homozygosity mapping for three affected siblings from families 5 and 7.

Each blue dot represents one individual marker or single-nucleotide polymorphism (SNP). For each SNP in the B allele frequency panels, a low B allele frequency indicates that the individual is a homozygote for the A allele; intermediate values mean they are heterozygotes; and a high B allele frequency means that they are homozygotes for the B allele.

Only one region >1.5Mb was found to be shared between all affected individuals of both families: chromosome 19 49,506,390-51,400,356bp (vertical blue area).

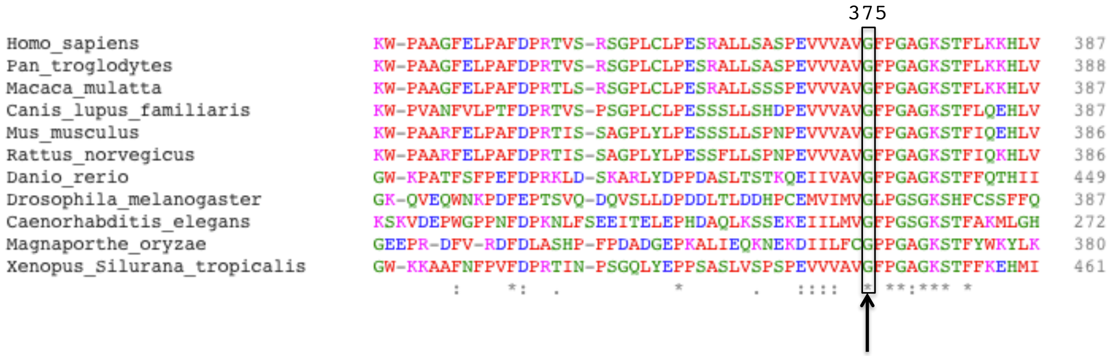

Figure S2. Conservation alignment for PNKP p.Gly375Trp variant.

The PNKP residue most commonly found to be mutated is conserved throughout evolution, as shown by alignment of the protein sequences of PNKP orthologues in various organisms with Clustal Omega software.

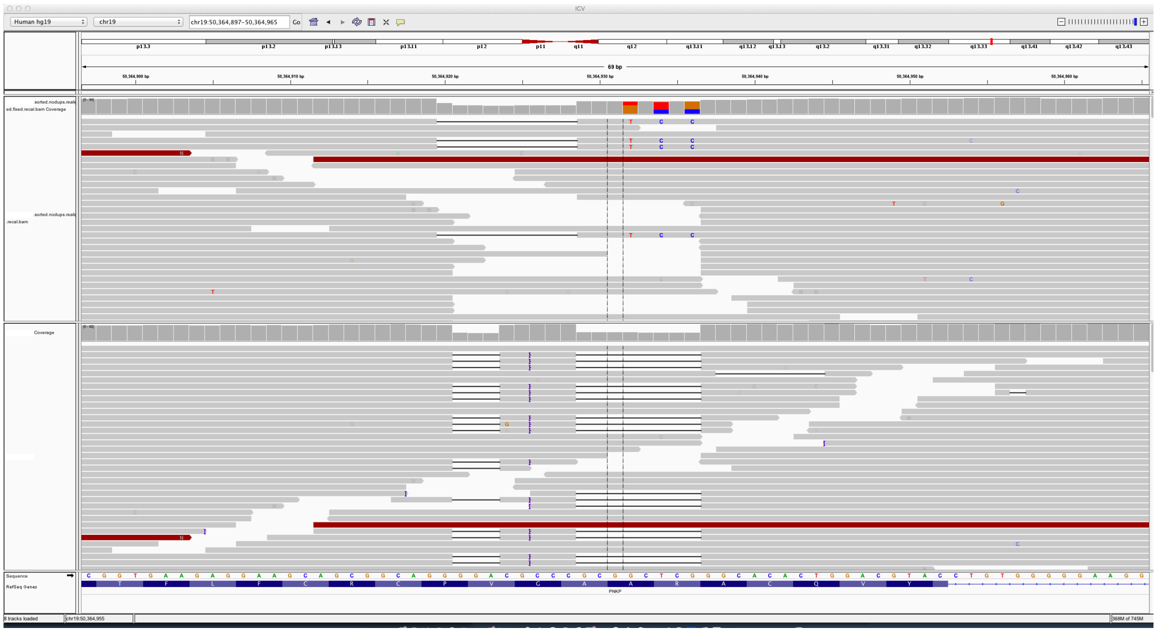

Figure S3. Visualization of the complex allele found in family 4.The proband in Family 4 was found to harbour a compound heterozygous mutation.
p.[(Thr408del)];[(Arg439Glyfs*52;Val443Serfs*25;Val443_Pro444delinsAla)] with the variant inherited from the father being a complex mutation. This variant was not properly classified when using a Bayesian genotype likelihood model (GATK UnifiedGenotyper) (top panel) and was only fully identified by using a local re-assembly of haplotypes in the

active region (GATK HaplotypeCaller v3.2-2) (bottom panel) as visualized in the Integrative Genomics Viewer (IGV) [1; 2].

Supplemental References

1. Thorvaldsdottir, H., Robinson, J.T., and Mesirov, J.P. (2013). Integrative Genomics Viewer (IGV): high-performance genomics data visualization and exploration. Brief Bioinform 14, 178-192.
2. Robinson, J.T., Thorvaldsdottir, H., Winckler, W., Guttman, M., Lander, E.S., Getz, G., and Mesirov, J.P. (2011). Integrative genomics viewer. Nat Biotechnol 29, 24-26.
